# Enhanced Selective Hydrogen Permeation through Graphdiyne Membrane: A Theoretical Study

**DOI:** 10.3390/membranes10100286

**Published:** 2020-10-15

**Authors:** Quan Liu, Long Cheng, Gongping Liu

**Affiliations:** 1 Analytical and Testing Center, Anhui University of Science and Technology, Huainan 232001, China; 2 State Key Laboratory of Materials-Oriented Chemical Engineering, College of Chemical Engineering, Nanjing Tech University, 30 Puzhu Road (S), Nanjing 211816, China; longcheng@njtech.edu.cn

**Keywords:** graphdiyne, molecular simulation, membrane separation, hydrogen purification

## Abstract

Graphdiyne (GDY), with uniform pores and atomic thickness, is attracting widespread attention for application in H_2_ separation in recent years. However, the challenge lies in the rational design of GDYs for fast and selective H_2_ permeation. By MD and DFT calculations, several flexible GDYs were constructed to investigate the permeation properties of four pure gas (H_2_, N_2_, CO_2_, and CH_4_) and three equimolar binary mixtures (H_2_/N_2_, H_2_/CO_2_, and H_2_/CH_4_) in this study. When the pore size is smaller than 2.1 Å, the GDYs acted as an exceptional filter for H_2_ with an approximately infinite H_2_ selectivity. Beyond the size-sieving effect, in the separation process of binary mixtures, the blocking effect arising from the strong gas–membrane interaction was proven to greatly impede H_2_ permeation. After understanding the mechanism, the H_2_ permeance of the mixtures of H_2_/CO_2_ was further increased to 2.84 × 10^5^ GPU by reducing the blocking effect with the addition of a tiny amount of surface charges, without sacrificing the selectivity. This theoretical study provides an additional atomic understanding of H_2_ permeation crossing GDYs, indicating that the GDY membrane could be a potential candidate for H_2_ purification.

## 1. Introduction

As an attractive alternative fuel source, hydrogen (H_2_) could eliminate the use of polluting fossil fuels in industry and transport in the future [1]. So, the H_2_ energy is critical to reduce global greenhouse gases and promote sustainable development because of its natural abundance and high efficiency of combustion [2,3]. Today, in many industrial and drilling streams, H_2_ is mainly produced from natural gas, hydro-electrolysis, and the combustion of hydrides [4,5]. However, these processes release several million tonnes of by-products (such as carbon dioxide, nitrogen, and methane) per year [6]. How to separate the target product of H_2_ from the undesirable species is crucial to improve production efficiency and reduce the cost. Traditional separation techniques, such as pressure swing adsorption and cryogenic separation, would consume a considerable amount of energy to collect H_2_ [7]. With the improved performances and lower operating conditions, the advanced membrane-based separation is seen as an alternative to significantly improve energy efficiency [8,9]. At room temperature and low transmembrane pressure, it is more instructive for experiments to develop membranes with both good H_2_ permeance and selectivity. [10]. 

Recently, two-dimensional (2D) carbon-based membranes have sparked global attention due to their atomic thickness [11]. Among various membranes, graphene-based materials are, assuredly, one of the powerful candidates for membrane separation according to our previous experimental [12,13] and simulation studies [14,15]. According to the separation mechanism of the size sieving effect, controllable pores in the 2D-material membrane are imperative for gas separation. However, it is not cost-effective to drill uniform pores in graphene, and this process may introduce non-selective defects in the membrane [16]. Alternatively, newly emerged graphdiyne (GDY) allotropes that formed by the periodic combination of sp and sp^2^ carbon atoms are proposed as alternative options for gas purification [16], because they process the well-distributed pores as well as the atomic membrane thickness [17]. Several studies have been reported to explore the gas permeation property through GDYs in recent years [18,19,20]. It is the comparable pores in GDYs that contribute to the outstanding gas separation properties, as Smith et al. first reported that the excellent H_2_ separation performance was ascribed to the small triangular pores in the GDYs [21]. The later reports also showed that the various sized pores in diverse GDYs could be employed to distinguish the different sized molecules, such as He [18], O_2_ [22], and CO_2_ [23,24], concluding that the size sieving effect dominated the transport mechanism of GDYs for gas separation.

Although the pore size is an important factor for gas separation membranes, other factors, particularly the surface properties, should not be neglected. Sang et al. [25] and Smith et al. [26] found that the functionalized surface of GDYs shown a better separation performance than that of the pristine one. Moreover, the adsorption phenomenon in GDYs also affected the permeation of gases, such as H_2_S [27] and CO_2_ [24,28]. It means, beyond the well-understood size sieving effect, there could be a more comprehensive mechanism to better describe gas transport through GDYs [19]. However, the main challenge is how such a mechanism determines the gas permeation properties, especially for selective H_2_ permeation through GDYs, and how it contributes to further improvement of the H_2_ permeance and selectivity of GDYs. In addition, most previous works focused on the first-principles calculations density functional theory (DFT) to study the selectivity of H_2_ over CO_2_, CO, N_2_, and CH_4_ [25,26,29]. A few studies calculated the H_2_ permeance by performing molecular dynamic (MD) simulations, which however were based on a rigid framework of GDYs [20,23,30] and might be too idealized for actual GDYs for gas separation. Moreover, it is unclear what the ultimate size of nanopores in GDYs is allowed to transport H_2_ molecules. Therefore, it is necessary to further understand the underlying separation mechanism of H_2_ purification through a carefully designed flexible GDY membrane that possesses both high H_2_ permeance and selectivity and find the ultimate diameter in GDYs for H_2_ permeation.

In this work, a series of 2D GDYs are computationally constructed to examine the permeation of four pure gases (H_2_, N_2_, CO_2_ and CH_4_) and their equimolar binary mixtures (H_2_/N_2_, H_2_/CO_2_ and H_2_/CH_4_). Assuming that the separation performance of H_2_ is affected by pore structures and surface charges, we systematically investigated these two effects by both MD and DFT calculations. Following the introduction, the atomic models of GDYs, as well as the separation systems, are illustrated in Section 2. In Section 3, the H_2_ permeance is calculated by the time evolution of the permeated molecules according to the MD results, and the ideal selectivity is evaluated by DFT calculations. The separation mechanism of H_2_ from binary mixtures is revealed by analyzing the diffusion coefficient, density contour, and energy barrier of the permeation. After that, the H_2_ permeance of the mixture of H_2_/CO_2_ is further improved by reducing the blocking effect. Finally, the concluding remarks are summarized in Section 4.

## 2. Models and Methods

**Figure 1:**
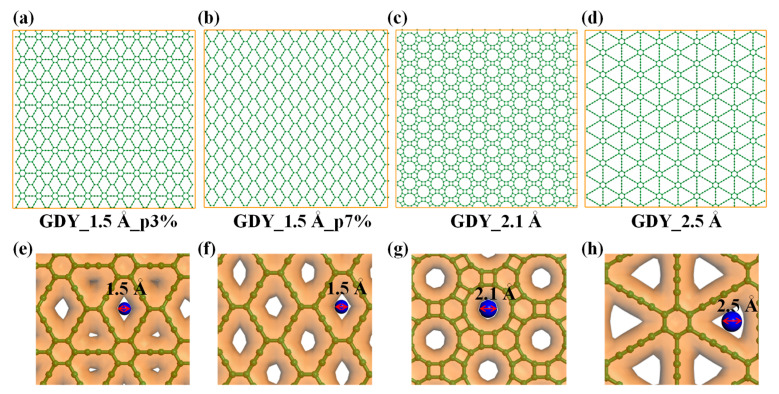
Membrane models. Atomic structures of graphdiyne (GDY) membranes with different pore structures: (**a**) GDY_1.5Å_p3%; (**b**) GDY_1.5Å_p7%; (**c**) GDY_2.1Å; and (**d**) GDY_2.5Å. The first two membranes have different porosities of 3% and 7%, respectively. (**e**–**h**) The criterion for the definition of the pore diameter in each GDY membranes.

The GDYs were constructed in the Material studio [31] and then subjected to geometry optimization in the Forcite module with 5000 iterations. As presented in Figure 1a–d, the dimensions of the membranes were 7.75 × 7.55 nm^2^ in this setup, and the investigated pore diameters were varied from 1.5 to 2.5 Å, which were measured according to the formula: *D =*$\;2\sqrt{A/\pi }$ by inserting a van der Waals sphere, where *A* is the open pore area, as depicted in Figure 1e–h. We noted that the membrane in Figure 1b coded as GDY_1.5Å_p7% has the porosity of 7%, which is larger than that of 3% in Figure 1a (GDY_1.5Å_p3%), although both of these two membranes have a similar pore diameter of 1.5 Å. The above-mentioned porosity was calculated by the formula *p =* $\frac{A_{pore}}{A_{mem}}$, where $A_{pore}$ and $A_{mem}$ denote to the areas of the totally unoccupied regions and the membrane surface, respectively.

**Figure 2:**
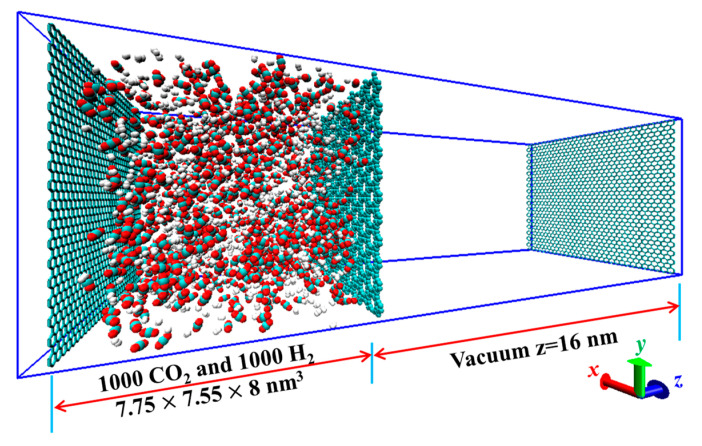
Simulation system of the equimolar binary mixture of H_2_/CO_2_ permeating through the GDY_2.1Å membrane. Atom: C (Cyan); O (red); H (white).

Based on the optimized membranes, MD calculations were performed to simulate the gas separation. Figure 2 shows the simulation system, where two chambers were isolated by the GDY membrane. The left chamber was a gas reservoir, comprising 2000 molecules of pure gases (or binary mixtures with the mixing ratio of 1000:1000, in volume ratio). The right one treated as a vacuum is the permeate side, which is the most common setting in MD simulations for collecting the permeated molecules. [20,23,30] At both ends of each chamber, rigid graphene was placed to prevent molecules from roaming between the periodic boxes. Considering that carbon-based membranes usually have good flexibility in experiments [32], all pores in GDYs were treated as flexible in our present work so that the atoms around pores could perform small displacements. Thus, the aperture would be enlarged to allow the passage of H_2_ even though the molecular size is larger than the pore size. On the contrary, the atoms on the edge of membranes were imposed with a position restriction to ensure the uniform lattice of GDYs and decrease the impact on gas permeation [33]. Furthermore, no collapse appeared in our system, suggesting good stability of the flexible structures. The framework of GDYs was described by the all-atom optimized potential. The four gases were represented by Lennard–Jones (LJ) and electrostatic potentials originating from our previous work [14]. Between dissimilar atoms, the interactions were assembled by the Lorentz–Berthelot combination rule [34].

In this study, MD simulations were all carried out using the GROMACS package (version 4.5.5) [35]. A static minimization with the steepest descent method was first carried out to remove the unreasonable contacts among each atom. Then, the separation system was pre-equilibrated in the isobaric–isothermal ensemble (constant temperature and constant pressure ensemble, named as NPT) for 2 ns only in the z-direction. Following this, another 20 ns MD simulations were performed in the canonical ensemble (constant temperature and constant volume ensemble, named as NVT) for data collection and further analysis, and the trajectories were stored every 1 ps. During the simulations, the classical equations of motion were integrated with a time step of 1 fs by using the leapfrog algorithm. The temperature was maintained at 300 K by coupling with the velocity rescaling [36] thermostats. The long-range electrostatic interactions were computed by using the method of particle mesh Ewald [37]. While the short-range van der Waals interactions were truncated at a cut-off distance of 1.2 nm, the periodic boundary conditions were implemented in all three directions. With the partial pressure gradient as the driving force [38], the gases would permeate through the GDYs. For a better understanding of the separation process, an animation is provided in the Supplementary Materials (Video S1).

## 3. Results and Discussion

### 3.1. Gas Permeation Behavior

The relationship between gas flux (J, mol·m^−2^·s^−1^) and permeance (S, mol·m^−2^·s^−1^·Pa^−1^) was described as Equation (1):

(1) $$J=\frac{dN}{A_{mem}N_{A}dt}=∆PS$$

where *N* refers to the number of permeated molecules, $N_{A}$ is the Avogadro constant, and ∆P is the transmembrane gas partial pressure estimated by Equation (2):

(2) $$∆P=\frac{(N_{0}-N_{ad}-N)kT}{V_{l}}-\frac{NkT}{V_{r}}$$

in which $N_{ad}$ refers to the number of gases adsorbed on the membrane surface, $V_{l}$ and $V_{r}$ are the volumes of the left and right chambers, respectively, and *k* represents the Boltzmann constant. Therefore, the time evolution of the number of permeated molecules is integrated as Equation (3), where *R* is the gas constant,

(3) $$N=\frac{(N_{0}-N_{ad})L_{r}}{\left(L_{l}+L_{r}\right)}\left(1-e^{-\frac{\mathrm{RT}S(L_{l}+L_{r})}{L_{l}L_{r}}t}\right)=\frac{2(N_{0}-N_{ad})}{3}\left(1-e^{-467.7St}\right).$$

**Figure 3:**
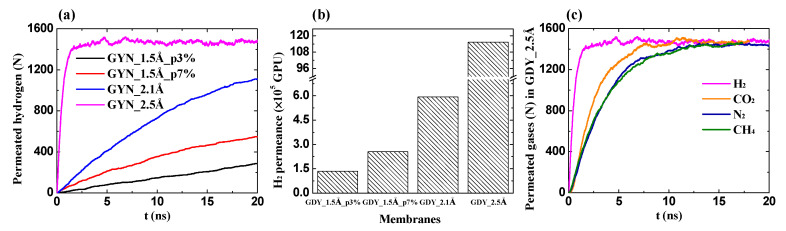
Pure gas permeation. (**a**) The time evolution of permeated H_2_ molecules; (**b**) The permeance of H_2_ through different GDYs; (**c**) The permeation of four gases in GDY_2.5Å.

As presented in Figure 3a, the number of permeated H_2_ is increased exponentially with the simulation time, which agrees well with the above mathematical analysis. The permeance of pure H_2_ is shown in Figure 3b. Obviously, it is remarkably enhanced with the increase of porosity and pore size. With the smallest pore of 1.5 Å, the porosity of 0.03 and 0.07 have the H_2_ permeance of 1.34 × 10^5^ and 2.55 × 10^5^ GPU (1 GPU = 3.35 × 10^−10^ mol·m^−2^·s^−1^·Pa^−1^), respectively. The permeation of other gases (i.e., CO_2_, N_2_ and CH_4_) through the GDYs was also simulated. It was shown that only the biggest pore with 2.5 Å allowed the passage of N_2_, CO_2_, and CH_4_, as presented in Figure 3c. The incompatibility of the kinetic diameter of gases (i.e., H_2_, 2.89 Å; N_2_, 3.64 Å; CO_2_, 3.30 Å; CH_4_, 3.8 Å) [39], and the pore size is ascribed to the flexible structures, implying that the membrane of GDY_2.5Å is not suitable for H_2_ purification. Moreover, the comparable pore with 2.1 Å diameter can not only completely block the other three gases but also process the good H_2_ permeance of 5.94 × 10^5^ GPU, which implies that the selectivities of H_2_ over CO_2_, N_2_, and CH_4_ can be extremely high in the membrane of GDY_2.1Å. We noted that the molecules of N_2_, CO_2_, and CH_4_ can not be detected in the permeation side as long as the pore diameter was smaller than 2.1 Å, indicating the infinitely low permeance of N_2_, CO_2_, and CH_4_.

**Figure 4:**
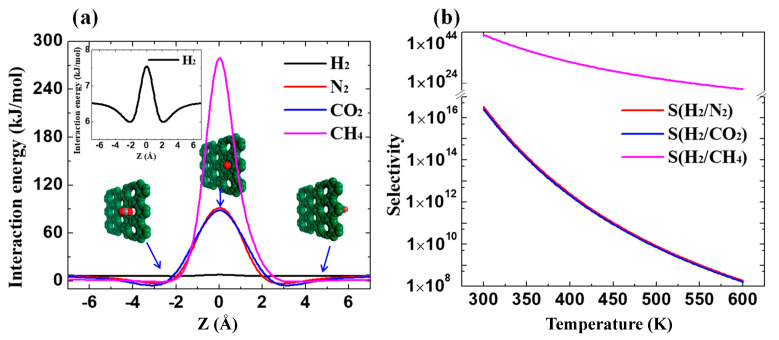
DFT calculations of gases crossing the membrane of GDY_2.1Å. (**a**) Minimum energy pathways of four gases. (**b**) Ideal selectivities of H_2_ over N_2_, CO_2_, and CH_4_ as functions of temperature.

To evaluate the ideal selectivities of H_2_ over other three gases in GDY_2.1 Å, the DFT calculations were performed to calculate permeation barriers as per our previous study [15]. Figure 4a illustrates the minimum energy pathway (MEP) of four gases crossing the membrane. The inset configurations are the energetically stable states of CO_2_ permeation at different locations. By searching the saddle point in MEPs, the energy barriers of permeation can be calculated. Evidently, the permeation barrier of H_2_ crossing the flexible GDY_2.1 Å is drastically reduced to 1.55 kJ/mol (Figure 4a, inset), which results in extraordinary H_2_ permeance. On the contrary, the permeation barriers of CO_2_, N_2_, and CH_4_ are all extremely high. In Figure 4b, the temperature-dependent H_2_ selectivity has an inverse correlation with the temperature according to the Arrhenius Equation (4), whereas *P_i_* is the permeation rate, *A* is the permeation prefactor that can be assumed as 10^11^ s^−1^ [38], and *T* is the temperature. For H_2_/CO_2_ and H_2_/N_2_, the ideal selectivities of H_2_ can be up to 10^17^ at 300 K, which is the same order with the modified GDYs [25]. It remains very high (>10^8^) even at 600 K, further suggesting the extraordinary H_2_ separation performance through GDY_2.1 Å.

(4) $$S_{i/j}=\frac{P_{i}}{P_{j}}=\frac{A_{i}exp\left(-\frac{E_{barrier,\;\;i}}{RT}\right)}{A_{j}exp\left(-\frac{E_{barrier,\;\;j}}{RT}\right)}$$

### 3.2. Transport Mechanism: Blocking Effect

**Figure 5:**
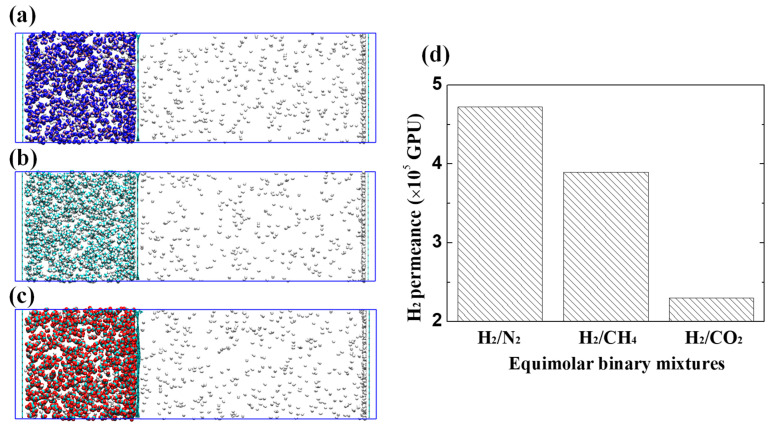
H_2_ permeation of three equimolar binary mixtures crossing the GDY_2.1Å. The final snapshots: (**a**) H_2_/N_2_, (**b**) H_2_/CH_4_, and (**c**) H_2_/CO_2_. Blue: N; cyan: C; red: O; white: H. (**d**) The H_2_ permeance of different binary mixtures.

For binary gas mixtures, the MD calculations were also performed to simulate the separation process. To visualize H_2_ purification properties through the membrane of GDY_2.1Å, the equilibrium configurations of final frames are presented in Figure 5a–c. As seen, the GDY_2.1Å membrane acts as an effective filter for H_2_ separation while completely blocking the passage of CO_2_, N_2_, and CH_4_, so that the H_2_ is largely gathering in the vacuum chamber. The corresponding H_2_ permeance of the three binary mixtures is presented in Figure 5d. Interestingly, the mixture of H_2_/N_2_ exhibits the highest H_2_ permeance of 4.71 × 10^5^ GPU, which is a little higher than that of H_2_/CH_4_ and almost twice as many as the mixture of H_2_/CO_2_. The primary reason is that on the membrane surface, the strongly interacting gas (CO_2_) preferentially adsorbs, blocking the transport pores of H_2_ molecules, thus resulting in a relatively low H_2_ permeance.

**Figure 6:**
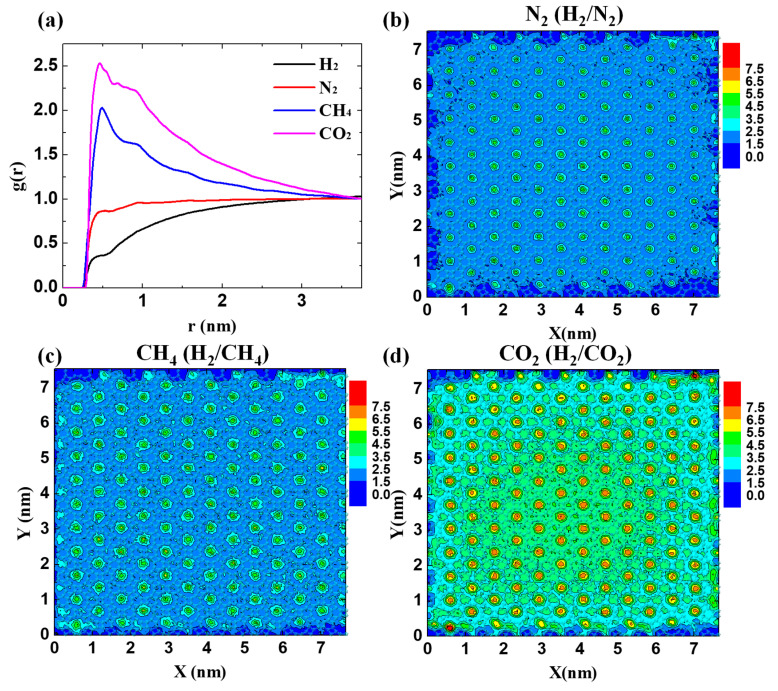
Blocking effect on the surface of GDY_2.1Å. (**a**) Radial distribution function (RDF) of gases around the membrane. Density contours of the impermeable gases on the surface: (**b**) N_2_ (H_2_/N_2_); (**c**) CH_4_ (H_2_/CH_4_); (**d**) CO_2_ (H_2_/CO_2_). The unit of density (N_w_/uc) is 1/(1.25Å^3^).

**Figure 7:**
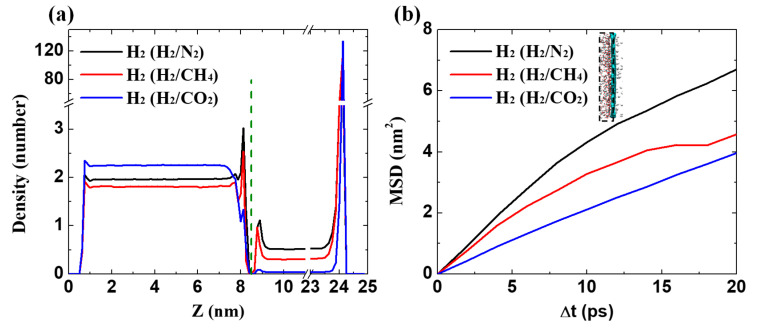
Permeation behavior of H_2_ in binary mixtures. (**a**) The number distributions along the z-direction in the last 10 ns. The membrane is located at the green dotted line; (**b**) The mean square displacement (MSD) curves of H_2_ crossing the GDY_2.1Å membrane. (Inset) The region within 0.5 nm of the membrane surface for MSD calculation.

Further analyses are carried out to understand this blocking effect. The radial distribution function (RDF) was calculated to present the affinity of GDY_2.1Å to four gases by using Equation (5):

(5) $$g_{ij}\left(r\right)=\frac{N_{ij}\left(r,\;r+∆r\right)V}{4\pi r^{2}∆rN_{i}N_{j}}$$

where *N_ij_* (*r*,*r* + Δ*r*) is the number of species *j* around *i* within a shell from r to *r* + Δ*r*, *r* is the distance between two species, and *N_i_* and *N_j_* refer to the numbers of atom types *i* and *j*, respectively. As presented in Figure 6a, there is an increasing trend of the gas–membrane interaction as H_2_ < N_2_ < CH_4_ < CO_2_. The weakest interaction together with the smallest molecular size endows H_2_ with exceptional permeance. Meanwhile, the strong interaction promotes the gases, particularly CO_2_ to preferentially adsorb on the membrane surface. To offer more intuitionistic information, the density contours of the distribution of N_2_, CH_4_, and CO_2_ were plotted on the GDY_2.1Å surface. The general distribution behavior of the three gases is similar as shown in Figure 6b–d, where the pores that are largely clogged by these gases are larger than the pore diameters. Nevertheless, the intensity of gas accumulation is quite different. As seen in Figure 6d, the pores are the most clogged, with the number of adsorbed CO_2_ molecules exceeding 6.5 N_w_/uc in every pore. Similar to the affinity analysis in Figure 6a, the extent of the blocking effect follows the increasing trend of N_2_ < CH_4_ < CO_2_, decreasing the placeholders of H_2_ on the membrane surface in Figure 7a.

According to the first peak in Figure 6a, the mean square displacement (MSD) of H_2_ molecules was analyzed within 0.5 nm of the membrane surface (Figure 7b, inset) from

(6) $$MSD\left(t\right)\;=\frac{1}{N}\langle \overset{N}{\underset{i=1}{\sum }}(r_{i}{\left(t\right)}^{2}-r_{i}{\left(t_{0}\right)}^{2})\rangle$$

(7) $$D=\;\frac{1}{6}\underset{t\to \infty }{lim}\frac{dMSD\left(t\right)}{dt}$$

where $r_{i}{\left(t\right)}^{2}-r_{i}{\left(t_{0}\right)}^{2}$ refers to the distance traveled by the atom (*i*) over the time interval of $t-t_{0}$. The diffusion coefficient (*D*) is determined by the linear slope of the MSD curve with Equation (7). The diffusion behavior in this confined region cannot sustain a normal movement with a large time interval, and it is sufficient to calculate the diffusion coefficient within 20 ps. The greater placeholders of H_2_, ascribing to the lower blocking effect of N_2_, promote the faster movement of H_2_, as presented in Figure 7b. H_2_ in three binary mixtures of H_2_/N_2_, H_2_/CH_4_, and H_2_/CO_2_ exhibits the diffusion coefficients of 0.57 × 10^−2^, 0.44 × 10^−2^, and 0.33 × 10^−2^ cm^2^/s, respectively. That is, the H_2_ permeation is indeed impeded by the preferentially adsorbed CO_2_ molecules. In other words, the GDY_2.1Å can separate H_2_ faster and more selectively from such a kind of binary mixture that the other species has a weak interaction toward GDY surfaces, such as H_2_/N_2_.

### 3.3. Surface Charge Effect

**Figure 8:**
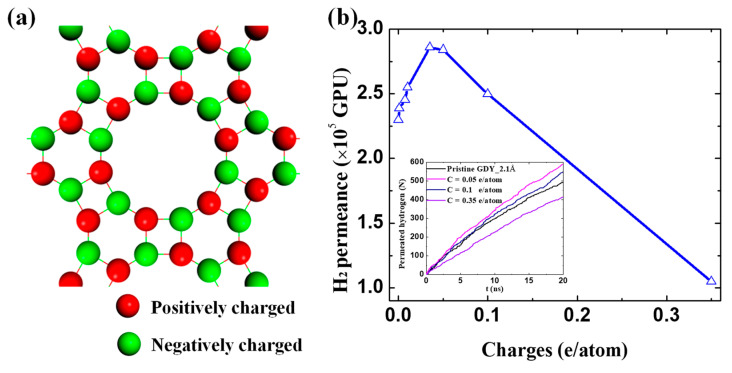
The effect of the surface charges on gas permeation. (**a**) The distribution of charges on the network of GDY_2.1Å; (**b**) H_2_ permeance of the binary mixture of H_2_/CO_2_ as a function of surface charges. (Inset) The time evolution of permeated H_2_ molecules through the charged GDYs.

Two important mechanisms dominated the gas separation. The first one is the size sieving effect, which favors the permeation of small-sized molecules of H_2_. While beyond the size sieving effect, in the separation process of binary mixtures, the blocking effect greatly impedes H_2_ permeation. The above understanding of the separation mechanism is beneficial to further guide the improvement of H_2_ permeance particularly for the mixture of H_2_/CO_2_ without sacrificing its selectivity. An effective way proposed here is to reduce the most severe blocking effect of CO_2_ by surface charge modification. As depicted in the inset figure in Figure 8a, the positive and negative charges are uniformly imposed on the network of GDY_2.1Å from ±0.00001 to ±0.35 e/atom, whereas the net charge of the whole membrane is zero. The CO_2_ molecules still cannot cross the membrane regardless of surface charges. Compared to the pristine GDY membrane, the increased H_2_ permeance of the binary mixture of H_2_/CO_2_ was observed when surface charges are lower than ±0.1 e/atom in Figure 8b. It can be up to 2.84 × 10^5^ GPU by imposing a tiny amount of surface charge of ±0.035–0.050 e/atom. This exceptionally high permeance is several orders of magnitude greater than the existed experiments [40,41,42], which is ascribed to the ultimate pore size and atomic thickness of GDYs. The following decreasing trend in Figure 8b was ascribed to the surface overcharge, where the generated strong electrostatic repels not only CO_2_ but also H_2_ from approaching. Thus, achieving the perfect balance between these two mechanisms, a tiny amount of surface charges is qualified to maximize the H_2_ permeance by reducing the blocking effect of strong interlaced molecules of CO_2_, meanwhile ensuring the placeholders of small-sized molecules of H_2_ as well.

## 4. Conclusions

In summary, a multiscale study combining MD and DFT calculations was performed to investigate the gas permeation through carefully designed flexible GDY membranes with different pore structures and surface charges. Four single gases and three equimolar binary mixtures (H_2_/other gas) were simulated to study the selective gas permeation through GDYs. Approximately infinite selectivities of H_2_ over N_2_, CO_2_, and CH_4_ in GDY_2.1Å membranes were demonstrated by DFT calculations. The underlying mechanism indicated that the blocking effect impeded H_2_ permeation and the GDY_2.1Å is prone to separate H_2_ from such binary mixtures in which the other species has a weak gas–membrane interaction. Moreover, by imposing a tiny amount of surface charges, the H_2_ permeance of the binary H_2_/CO_2_ mixture was further enhanced up to 2.84 × 10^5^ GPU without sacrificing the selectivity. These excellent transport properties make the GDYs a promising candidate for efficient H_2_ purification. Although the present work is a theoretical study, it is believable that the GDYs can be elaborately designed for realistic separations with the improvement of a well-controlled synthesis strategy.

## Supplementary Materials

The following are available online at https://www.mdpi.com/2077-0375/10/10/286/s1, Video S1: The separation process of the binary mixture of H_2_/CO_2_ through GDY_2.1Å.
